# Levels of chronic systemic inflammation markers in patients with multi-stages of esophageal and gastric lesions

**DOI:** 10.3389/fonc.2026.1751801

**Published:** 2026-07-08

**Authors:** Yanyan Li, Qiaofen Lin, Xiaoyin Huang, Jiamin Gong, Ruimei Feng, WanXin Li, Heng He, Wei Liang, Shanshan Du, Jun Chen, Jianhui Song, Weimin Ye

**Affiliations:** 1 Institute of Population Medicine, Fujian Medical University, Fuzhou, Fujian, China; 2 Department of Epidemiology and Health Statistics, School of Public Health, Fujian Medical University, Fuzhou, Fujian, China; 3 Department of Epidemiology, School of Public Health, Shanxi Medical University, Taiyuan, Shanxi, China; 4 Department of Digestive Endoscopy, Shengli Clinical Medical College of Fujian Medical University, Fujian Provincial Hospital, Fuzhou, Fujian, China; 5 Digestive Endoscopy Center, Putian Hospital of Traditional Chinese Medicine, Putian, Fujian, China; 6 Department of Medical Epidemiology and Biostatistics, Karolinska Institutet, Stockholm, Sweden

**Keywords:** case-control study, chronic systemic inflammation, esophageal cancer, gastric cancer, multistage lesions

## Abstract

**Background:**

Chronic systemic inflammation (CSI) is linked to esophageal and gastric cancer (EC and GC) risk. However, few studies have explored the potential role of CSI markers in the development of EC/GC, from normal mucosa, benign lesion to carcinomatous degeneration.

**Methods:**

This case-control study was based on a population-based EC&GC screening program in a high-incidence area of China. For esophageal lesions, 633 participants from groups with normal mucosa (E-con, N = 202), benign lesion (E-BL, N = 229), and precancerous lesion/cancer (E-case, N = 202, matched with E-con) were included. For gastric lesions, 156 pairs of samples from four groups with normal/superficial gastritis (G-con), atrophic gastritis (AG), intestinal metaplasia (IM), and precancerous lesion/cancer (G-case) were matched. Multivariable logistic regression was used to explore the association between CSI markers and esophageal/gastric lesions.

**Results:**

Among 13 CSI indicators, E-case group showed a slightly lower mean platelet volume (MPV) level than E-con or E-BL group. In logistic regression models, MPV was negatively associated with E-case risk compared to E-con (OR = 0.65, 95%CI: 0.46-0.92) or (E-con+E-BL) group (OR = 0.81, 95%CI: 0.68-0.97). The levels of 13 CSI markers were similar across four gastric lesion groups. However, lower MPV was associated with an increased risk of AG (OR = 0.70, 95%CI: 0.50-0.97), IM (OR = 0.62, 95%CI: 0.45-0.86), G-case (OR = 0.56, 95%CI: 0.37-0.86), and all three lesion (AG+IM+G-case, OR = 0.69, 95%CI: 0.52-0.93) compared to G-con group.

**Conclusion:**

MPV was negatively associated with the risk of esophageal precancerous lesion/cancer, and also likely associated with an decreased risk of AG and more severe lesions in Correa’s cascade of pathological process of GC.

## 1 Introduction

Inflammation plays a crucial role in the physiologic and pathologic processes of “healing” a wound or infection, involving a self-limited multifactorial network of pro-inflammatory cytokines and anti-inflammatory signals. Persistence of initiating factors or unresolved inflammatory responses often lead to chronic systemic inflammation (CSI), which is associated with an increased risk of various metabolic diseases (1). The role of inflammation in cancer initiation, progression and prognosis has long been reported (2, 3), with several CSI markers recommended as early biomarkers for cancer recognition (2). In upper gastrointestinal cancer (UGC), systemic inflammation response index (SIRI) (4), albumin (ALB) and lymphocyte-to-monocyte ratio (LMR) (5) have been reported to be related to the prognosis and overall survival of esophageal cancer (EC) patients. Systemic immune-inflammation index (SII) (6, 7), platelet/lymphocyte ratio (PLR) (8), and SIRI (9) are associated with survival outcomes in gastric cancer (GC), while SII and neutrophil-to-lymphocyte ratio (NLR) influence the treatment response in GC (10, 11). All these results indicate an important role of CSI in EC and GC. However, most studies focused on the prognosis of clinically diagnosed cancer patients, and studies on multistage histological lesions of the esophageal or gastric mucosa are scarce. It is unclear whether and how CSI acts from normal mucosa, benign lesion, to carcinomatous degeneration.

Both GC and EC have brought heavy burden to public health worldwide, and about half of the cases were reported in China (12, 13). Fujian Province, located in the southeast coastal region of China, is a high incidence and mortality area of UGC (14). Therefore, a natural population-based UGC screening program was initiated in Putian City, Fujian Province, to promote the early diagnosis of UGC and explore potential risk factors and biomarkers for UGC (15). Based on this program, case-control studies were conducted, which covered the population with multi-stage lesions during EC and GC progression. In this study, we reviewed the currently reported CSI indicators and applied them to the case-control study, trying to investigate the potential role of CSI in the development of EC and GC.

## 2 Materials and methods

### 2.1 Study population

**Figure 1:**
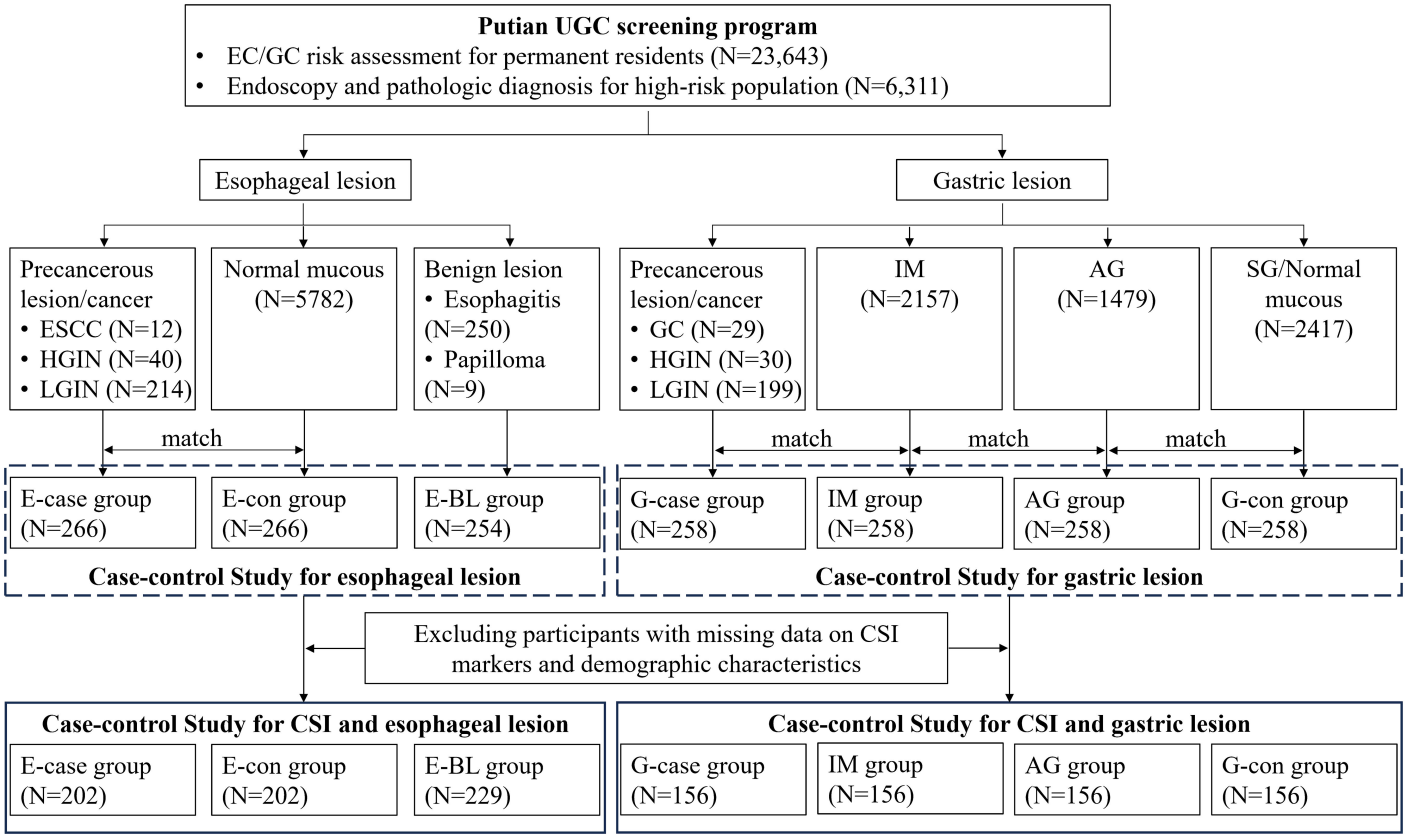
The flow chart of study design and population selection in current Study.

The Putian UGC screening program was conducted from June 2020 to June 2023 in Putian City, Fujian Province, Southeast China, and recruited permanent residents aged 40 to 80 years for EC/GC risk assessment (N = 23,643). Among them, a total of 6,311 participants underwent endoscopy and pathological diagnosis. In the diagnosis of esophageal diseases, it covered normal (N = 5782), esophagitis (N = 250), papilloma (N = 9), low (LGIN, N = 214)/high- (LGIN, N = 40) grade intraepithelial neoplasia and esophageal squamous cell carcinoma (ESCC, N = 12). And 4 participants whose tissue samples were too small to undergo pathological diagnosis were excluded in analysis of esophageal lesion. In the stomach, diagnoses included normal/superficial gastritis (normal and SG, N = 2417), atrophic gastritis (AG, N = 1479), intestinal metaplasia (IM, N = 2157), LGIN (N = 199), HGIN (N = 30), and GC (N = 29). Subsequently, case-control studies on lesions in the esophagus and stomach were designed. For the esophagus, LGIN/HGIN/ESCC patients were included in case group (E-case, N = 266), and all esophagitis and papilloma (N = 254) were included in esophageal benign lesion group (E-BL). Then, control group (E-con) was matched 1:1 by age and sex with the E-case group among participants with normal mucosa. Based on Correa’s cascade pathway of progression to GC, gastric LGIN/HGIN/GC patients (N = 258) were included in case group (G-case), then subgroups of IM, AG, and SG/normal (G-con) were matched 1:1 by age and sex. In current study, after excluding participants without CSI indicators and demographic characteristics, 202, 229, and 202 participants from E-case, E-BL, and E-con groups were included in the analysis of the association between CSI and esophageal lesion. Meanwhile, 156 pairs of matched participants from G-case, IM, AG, and G-con groups were included in the analysis of gastric lesion. The process flow chart is shown in **Figure 1**.

The study protocol was approved by the Ethics Committee of Fujian Province (approval number: 2020-73 (from July, 2020 to March, 2021), 2021-112 (from April, 2021 to June, 2022), 2022-86 (from July, 2022 to December, 2024), and 2024-275 (the updated version of biological sample testing from Putian UGC screening program)). Each participant voluntarily signed a paper informed consent form in person prior to the physical examination.

### 2.2 Data collection

Face-to-face interviews were conducted by trained interviewers using a structured electronic questionnaire to collect information from participants, including socio-demographic characteristics, lifestyle variables (smoking, alcohol consumption, and physical activity), disease history, and medication history. The electronic questionnaire was developed independently by the research team (https://cohort.fjmu.edu.cn/co-cas/login). Height and weight measurements were taken by trained staff, and body mass index (BMI) was calculated as weight (kilograms) divided by the square of height (meters). Fasting blood samples were collected from all participants after a fasting period of at least 8 hours. Albumin (ALB) levels were measured using an automatic analyzer (Toshiba, Tokyo, Japan). White blood cells (WBC), lymphocytes (LYMPH), neutrophils (NEUT), monocytes (MONO), platelets (PLT), and mean platelet volume (MPV) were measured using an automated hematology analyzer (SYSMEX, Kyoto, Japan).

### 2.3 Endoscopy and pathologic diagnosis

The endoscopic examination included plain white light endoscopy, electron-stained endoscopy, and iodine-stained vein endoscopy for the observation of esophageal and gastric lesions. The examination was conducted in accordance with clinical endoscopy quality control requirements for continuous, sequential and standardized image collection, and tissue samples were taken from suspected esophageal lesions for histopathological diagnosis by professional pathologists. Pathological diagnostic criteria referred to the Chinese Expert Consensus Opinion on Early Esophageal Cancer Screening and Endoscopic Diagnosis and Treatment.

The diagnostic results of esophageal lesions included esophageal mucosal epithelial inflammation (Esophagitis), papilloma, LGIN, HGIN and ESCC. The histopathological criteria are: (1) Esophageal mucosal epithelial inflammation: lymphocyte and plasma cell infiltration, basal cell hyperplasia (>15% of the mucosal thickness). (2) Esophageal papilloma: exophytic papillary or verrucous protrusion, with a branched fibrovascular core under the microscope, and the surface covered with squamous epithelium, with well-differentiated cells. (3) LGIN: disordered epithelial layers, basal cell hyperplasia extending to the lower 1/3 of the mucosa. (4) HGIN: disordered epithelial layers throughout, basal cell hyperplasia extending to the upper 2/3 or the entire layer. (5) ESCC: ① Intramucosal carcinoma (T1a): breaks through the basement membrane but is limited to the mucosal layer; ② Submucosal invasive carcinoma (T1b and above): invades the submucosa or deeper.

For the stomach, mucosal specimens were taken from gastric body, antrum, and suspected lesions. The diagnostic results of gastric lesions included SG, AG, IM, LGIN, HGIN, and GC, based on the classification criteria from the World Health Organization (WHO) (16) and the Updated Sydney System for the classification and grading of gastritis (17). The diagnostic criteria are: (1) SG: lymphocyte and plasma cell infiltration (≥5 per high-power field or increased density in the lamina propria), which may be accompanied by neutrophil activity. Glandular structure is intact: no atrophy or IM. (2) AG: Glandular reduction: reduction of intrinsic glands ≥1/3. (3) IM: goblet cells replace the native gastric glands. (4) LGIN: limited dysplasia: mucosal submucosa 1/3 layer, mild glandular structure disorder. Cellular atypia: nuclear enlargement (≤2 times normal), partial loss of polarity. (5) HGIN: sieve-shaped/papillary/back-to-back glandular mucosa submucosa 2/3 layer or above involved, nuclear ≥3 times normal, loss of polarity, increased mitotic figures. p40/p63 staining shows intact basement membrane (11). GC: ① Intramucosal carcinoma (T1a): breaks through the basement membrane but is limited to the mucosal layer; ② Submucosal invasive carcinoma (T1b and above): invades the submucosa or deeper.

### 2.4 CSI indicators

A total of 13 indicators were used to assess the level of CSI in the population. Of these, WBC, LYMPH, NEUT, MONO, MPV and ALB were obtained by laboratory measurements, while the remaining seven indicators were calculated according to previously reported equations. The normal reference ranges of 6 measured indicators and equations of 7 calculated indicators were shown below:

WBC: 3.5-9.5 10^9^/L
LYMPH: 1.1-3.2 10^9^/L
NEUT: 1.8-6.3 10^9^/L
MONO: 0.1-0.6 10^9^/L
MPV: 9.1-12.1 fL
ALB: 35–52 g/L
NLR=NEUT/lymphocyte (LYMPH) (18)
Derived NLR (d_NLR)=NEUT/(WBC−NEUT) (18)
PLR=PLT/LYMPH (18)
Lymphocyte−monocyte ratio (LMR)=LYMPH/monocytes (18)
SII =PLT × NEUT/LYMPH (19)
Advanced lung cancer inflammatory index (ALI)=BMI × ALB/NLR (20)
SIRI=NEUT × MONO/LYMPH (21)

### 2.5 Statistical analysis

Statistical analysis was performed using SAS (Cary, NC, version 9.4), with a two-sided *P* value < 0.05 considered as statistically significant. Based on data distribution, continuous variables were expressed as mean ± standard deviation (SD) or median M (P25, P75). Prior to analysis, normality of the data was assessed using the Shapiro-Wilk test. The Wilcoxon Rank-Sum Test (WRST) was performed to compare two independent samples, and the Kruskal-Wallis H test was performed for multiple independent samples. Cohen’s d values were tested to measure how large the difference between two groups. Generalized estimating equations were used for between-group analysis for two and multiple matched samples. Categorical information was described by frequency n (percentage %) and compared using the Chi-squared test. Conditional logistic regression models were constructed to investigate the association between CSI and lesions for matched case-control studies, while unconditional logistic regression was applied for unmatched groups. All CSI indicators are Z-standardized before regression analysis. The Z-standardization formula used in “PROC STANDARD” is:


$$\text{Ζ}=\frac{\chi -\bar{X}}{S}$$

χ=original variable value
$\bar{X}$=sample mean of that variable (automatically calculated by PROC STANDARD)
S=sample standard deviation of that variable (automatically calculated by PROC STANDARD, using n-1 denominator)

Odd ratios (ORs) and 95% confidence intervals (CIs) were calculated to assess the relative risk for esophageal/gastric lesions. Model 1 was unadjusted. Model 2 was adjusted for age and sex. Model 3 was further adjusted for age, sex, alcohol drinking status, smoking status, education, marriage, occupation, BMI, family wealth score, physical activity, and family history of cancer, selected according to the clinical priority principle. *Helicobacter pylori (H. pylori)* infection status were additionally adjusted in model 3 for gastric lesion analysis. All analyses were conducted on esophageal or gastric lesions respectively.

## 3 Results

### 3.1 The demographic characteristics of study population

In case-control study on esophageal lesions, a total of 633 participants were included. Their demographic characteristics are described in **Table 1**. The mean age of the population was 64.19 ± 6.56 years, and 42.65% were males. Among them, 67.93% of the population were illiteracy and primary school education, while 20.38% and 9.64% were current smokers and alcohol drinkers, respectively. In family disease history, 25.91% of the population reported a history of cancer among first-degree relatives. In this study, 202 participants were identified with precancerous lesions/cancer (E-case). The E-case group tended more likely to be farmers and have higher levels of physical activity than E-BL and E-con groups.

**Table 1:** The demographic characteristics of study population with esophageal lesions at different stages.

Characteristics	Total	E-case	E-BL	E-con
Participants, N	633	202	229	202
Age, years	64.19 ± 6.56	64.60 ± 6.25	63.40 ± 7.08	64.67 ± 6.17
(40,50]	18(2.84)	4(1.98)	11(4.80)	3(1.49)
(50,60]	116(18.33)	34(16.83)	50(21.83)	32(15.84)
(60,70]	357(56.40)	115(56.93)	121(52.84)	121(59.90)
(70,80]	142(22.43)	49(24.26)	47(20.52)	46(22.77)
Sex, N(%)				
Male	270(42.65)	91(45.05)	88(38.43)	91(45.05)
Female	363(57.35)	111(54.95)	141(61.57)	111(54.95)
BMI, kg/m^2^	24.47 ± 3.30	24.07 ± 3.34	24.89 ± 3.13	24.41 ± 3.40
Underweight	19(3.00)	8(3.96)	4(1.75)	7(3.47)
Normal	253(39.97)	90(44.55)	81(35.37)	82(40.59)
Overweight	272(42.97)	81(40.10)	108(47.16)	83(41.09)
Obesity	89(14.06)	23(11.39)	36(15.72)	30(14.85)
Education, N(%)				
Illiteracy	200(31.60)	66(32.67)	76(33.19)	58(28.71)
Primary school	230(36.33)	76(37.62)	83(36.24)	71(35.15)
Junior high school	128(20.22)	38(18.81)	38(16.59)	52(25.74)
High school and above	75(11.85)	22(10.89)	32(13.97)	21(10.40)
Occupation, N(%)				
Farmer	116(18.33)	47(23.27)	34(14.85)	35(17.33)
Worker	103(16.27)	29(14.36)	36(15.72)	38(18.81)
Others	414(65.40)	126(62.38)	159(69.43)	129(63.86)
Marital status, N(%)				
Unmarried	0	0	0	0
Married	554(87.52)	178(88.12)	211(92.14)	165(81.68)
Divorced/widowed	79(12.48)	24(11.88)	18(7.86)	37(18.32)
Family wealth score, N(%)				
Q1-lowest	403(63.67)	124(61.39)	148(64.63)	131(64.85)
Q2	83(13.11)	23(11.39)	30(13.10)	30(14.85)
Q3	88(13.90)	27(13.37)	38(16.59)	23(11.39)
Q4	6(0.95)	2(0.99)	2(0.87)	2(0.99)
Q5	53(8.37)	26(12.87)	11(4.80)	16(7.92)
Smoking status, N(%)				
Never	439(69.35)	133(65.84)	169(73.80)	137(67.82)
Previous	65(10.27)	24(11.88)	24(10.48)	17(8.42)
Current	129(20.38)	45(22.28)	36(15.72)	48(23.76)
Alcohol status, N(%)				
Never	537(84.83)	172(85.15)	196(85.59)	169(83.66)
Previous	35(5.53)	8(3.96)	12(5.24)	15(7.43)
Current	61(9.64)	22(10.89)	21(9.17)	18(8.91)
Physical activity, MET/day	23.12 ± 17.53	24.53 ± 18.42	22.10 ± 16.14	22.85 ± 18.12
≤ 12.99	214(33.81)	67(33.17)	78(34.06)	69(34.16)
(12.99,24.59]	187(29.54)	52(25.74)	72(31.44)	63(31.19)
>24.59	232(36.65)	83(41.09)	79(34.50)	70(34.65)
First-degree family history of cancer, N(%)				
Yes	164(25.91)	53(26.24)	69(30.13)	42(20.79)
No	443(69.98)	138(68.32)	152(66.38)	153(75.74)
Unknown	26(4.11)	11(5.45)	8(3.49)	7(3.47)

E-con was matched group with E-case group by age and sex.

In case-control study on gastric lesions, a total of 624 participants were included, of which 156 were identified with G-case, IM, AG, and G-con, respectively (**Table 2**). In this study, G-case group also had a higher proportion of farmers, but lower levels of physical activity than other lesions or G-con group.

**Table 2:** The demographic characteristics of study population with gastric lesions at different stages.

Characteristics	Total	G-case	IM	AG	G-con
Participant N	624	156	156	156	156
Age, years	63.57 ± 7.04	63.60 ± 7.10	63.60 ± 7.05	63.51 ± 7.02	63.58 ± 7.06
(40,50]	28(4.49)	7(4.49)	7(4.49)	7(4.49)	7(4.49)
(50,60]	120(19.23)	30(19.23)	30(19.23)	30(19.23)	30(19.23)
(60,70]	345(55.29)	86(55.13)	86(55.13)	87(55.77)	86(55.13)
(70,80]	131(20.99)	33(21.15)	33(21.15)	32(20.51)	33(21.15)
Sex, N(%)					
Male	412(66.03)	103(66.03)	103(66.03)	103(66.03)	103(66.03)
Female	212(33.97)	53(33.97)	53(33.97)	53(33.97)	53(33.97)
BMI, kg/m^2^	24.37 ± 3.17	24.33 ± 3.28	24.28 ± 3.14	24.68 ± 3.15	24.20 ± 3.11
Underweight	12(1.92)	2(1.28)	5(3.21)	2(1.28)	3(1.92)
Normal	278(44.55)	67(42.95)	72(46.15)	61(39.10)	78(50.00)
Overweight	249(39.90)	67(42.95)	54(34.62)	71(45.51)	57(36.54)
Obesity	85(13.62)	20(12.82)	25(16.03)	22(14.10)	18(11.54)
Education, N(%)					
Illiteracy	125(20.03)	38(24.36)	33(21.15)	24(15.38)	30(19.23)
Primary school	240(38.46)	64(41.03)	61(39.10)	58(37.18)	57(36.54)
Junior high school	172(27.56)	38(24.36)	37(23.72)	56(35.90)	41(26.28)
High school and above	87(13.94)	16(10.26)	25(16.03)	18(11.54)	28(17.95)
Occupation, N(%)					
Farmer	91(14.58)	27(17.31)	24(15.38)	23(14.74)	17(10.90)
Worker	116(18.59)	24(15.38)	25(16.03)	32(20.51)	35(22.44)
Others	417(66.83)	105(67.31)	107(68.59)	101(64.74)	104(66.67)
Marital status, N(%)					
Unmarried	0	0	0	0	0
Married	557(89.26)	140(89.74)	138(88.46)	147(94.23)	132(84.62)
Divorced/widowed	67(10.74)	16(10.26)	18(11.54)	9(5.77)	24(15.38)
Family wealth score, N(%)					
Q1-lowest	427(68.43)	111(71.15)	107(68.59)	111(71.15)	98(62.82)
Q2	72(11.54)	14(8.97)	19(12.18)	18(11.54)	21(13.46)
Q3	85(13.62)	20(12.82)	23(14.74)	17(10.90)	25(16.03)
Q4	6(0.96)	2(1.28)	2(1.28)	1(0.64)	1(0.64)
Q5	34(5.45)	9(5.77)	5(3.21)	9(5.77)	11(7.05)
Smoking status, N(%)					
Never	318(50.96)	76(48.72)	76(48.72)	90(57.69)	76(48.72)
Previous	114(18.27)	28(17.95)	28(17.95)	35(22.44)	23(14.74)
Current	192(30.77)	52(33.33)	52(33.33)	31(19.87)	57(36.54)
Physical activity, MET/day					
Never	455(72.92)	116(74.36)	116(74.36)	109(69.87)	114(73.08)
Previous	58(9.29)	10(6.41)	14(8.97)	17(10.90)	17(10.90)
Current	111(17.79)	30(19.23)	26(16.67)	30(19.23)	25(16.03)
Physical activity, MET/day	21.68 ± 17.06	18.40 ± 13.33	21.47 ± 16.90	24.54 ± 18.38	22.30 ± 18.68
≤ 12.99	235(37.66)	70(44.87)	60(38.46)	42(26.92)	63(40.38)
(12.99,24.59]	197(31.57)	49(31.41)	48(30.77)	57(36.54)	43(27.56)
>24.59	192(30.77)	37(23.72)	48(30.77)	57(36.54)	50(32.05)
First-degree family history of cancer, N(%)					
Yes	147(23.56)	30(19.23)	45(28.85)	41(26.28)	31(19.87)
No	450(72.12)	119(76.28)	105(67.31)	111(71.15)	115(73.72)
Unknown	27(4.33)	7(4.49)	6(3.85)	4(2.56)	10(6.41)

IM, AG, and G-con were matched groups with G-case group by age and sex.

### 3.2 The association between CSI and esophageal lesion

Across the 13 CSI indicators, E-con and E-BL groups tended to exhibit slightly higher levels of WBC, LYMPH, NEUT, MPV, LMR, and ALI than E-case group, but without reaching statistical significance. When compared in pairs, MPV was higher in E-con and E-BL groups than in E-case group, with marginal *P*-values of 0.086 and 0.055 with corresponding Cohen’s d values of 0.087and 0.195, and LMR of E-BL group was significantly higher than that of E-con (*P* = 0.049, Cohen’s d=0.203) and E-case groups (*P* = 0.037, Cohen’s d=0.206) (**Table 3**).

**Table 3:** The chronic systemic inflammation levels of different stages of esophageal lesions.

CSI indicators	Overall (N = 633)	E-con (N = 202)	E-BL (N = 229)	E-case (N = 202)	*P* value	P value of subgroup analysis					
						E-con vs E-BL		E-con vs E-case		E-BL vs E-case	
						*P* value	Cohen’s d	*P* value	Cohen’s d	*P* value	Cohen’s d
WBC, 109/L	6.00(5.10,7.10)	5.97(5.05,6.86)	6.10(5.23,7.25)	5.92(5.00,7.12)	0.460	0.280	0.079	0.972	0.021	0.292	0.102
LYMPH, 109/L	2.06(1.70,2.44)	2.05(1.70,2.38)	2.10(1.73,2.51)	2.04(1.61,2.48)	0.189	0.113	0.167	0.938	0.031	0.124	0.128
NEUT, 109/L	3.35(2.70,4.15)	3.28(2.70,4.23)	3.42(2.74,4.13)	3.25(2.70,4.15)	0.725	0.567	0.051	0.872	0.020	0.443	0.071
MONO, 109/L	0.36(0.29,0.44)	0.36(0.29,0.45)	0.35(0.29,0.43)	0.36(0.28,0.45)	0.729	0.456	0.091	0.911	0.019	0.549	0.105
MPV, fL	10.60(9.80,11.40)	10.65(9.80,11.40)	10.70(9.80,11.50)	10.40(9.70,11.10)	0.111	0.880	0.010	0.086	0.087	0.055	0.195
ALB, g/L	47.40(45.40,49.20)	47.60(45.50,49.20)	47.20(45.50,49.20)	47.45(45.30,49.10)	0.695	0.613	0.044	0.401	0.107	0.700	0.064
NLR	1.61(1.27,2.10)	1.62(1.30,2.12)	1.59(1.26,2.02)	1.65(1.25,2.17)	0.623	0.365	0.078	0.924	0.012	0.454	0.086
d_NLR	1.29(1.03,1.63)	1.30(1.05,1.63)	1.28(1.04,1.59)	1.29(1.02,1.67)	0.808	0.537	0.077	0.870	0.019	0.640	0.052
PLR	113.94(89.55,138.63)	116.53(91.67,137.93)	109.27(85.76,133.80)	116.18(94.15,146.35)	0.110	0.168	0.165	0.535	0.074	0.040	0.237
LMR	5.89(4.67,7.21)	5.72(4.45,7.04)	6.23(4.96,7.38)	5.60(4.48,7.08)	0.060	0.049	0.203	0.917	0.004	0.037	0.206
SII	373.27(280.02,507.65)	377.96(280.00,513.01)	367.11(274.31,484.24)	386.51(286.74,520.97)	0.555	0.404	0.048	0.812	0.053	0.322	0.097
SIRI	0.57(0.42,0.82)	0.60(0.43,0.88)	0.55(0.42,0.73)	0.57(0.41,0.84)	0.382	0.170	0.110	0.743	0.005	0.360	0.113
ALI	708.64(527.17,919.71)	706.07(528.95,862.19)	735.35(564.20,935.16)	645.57(498.01,945.21)	0.237	0.204	0.086	0.697	0.005	0.116	0.079

Data are presented as median with the interquartile range [M (P25-P75)].

**Figure 2:**
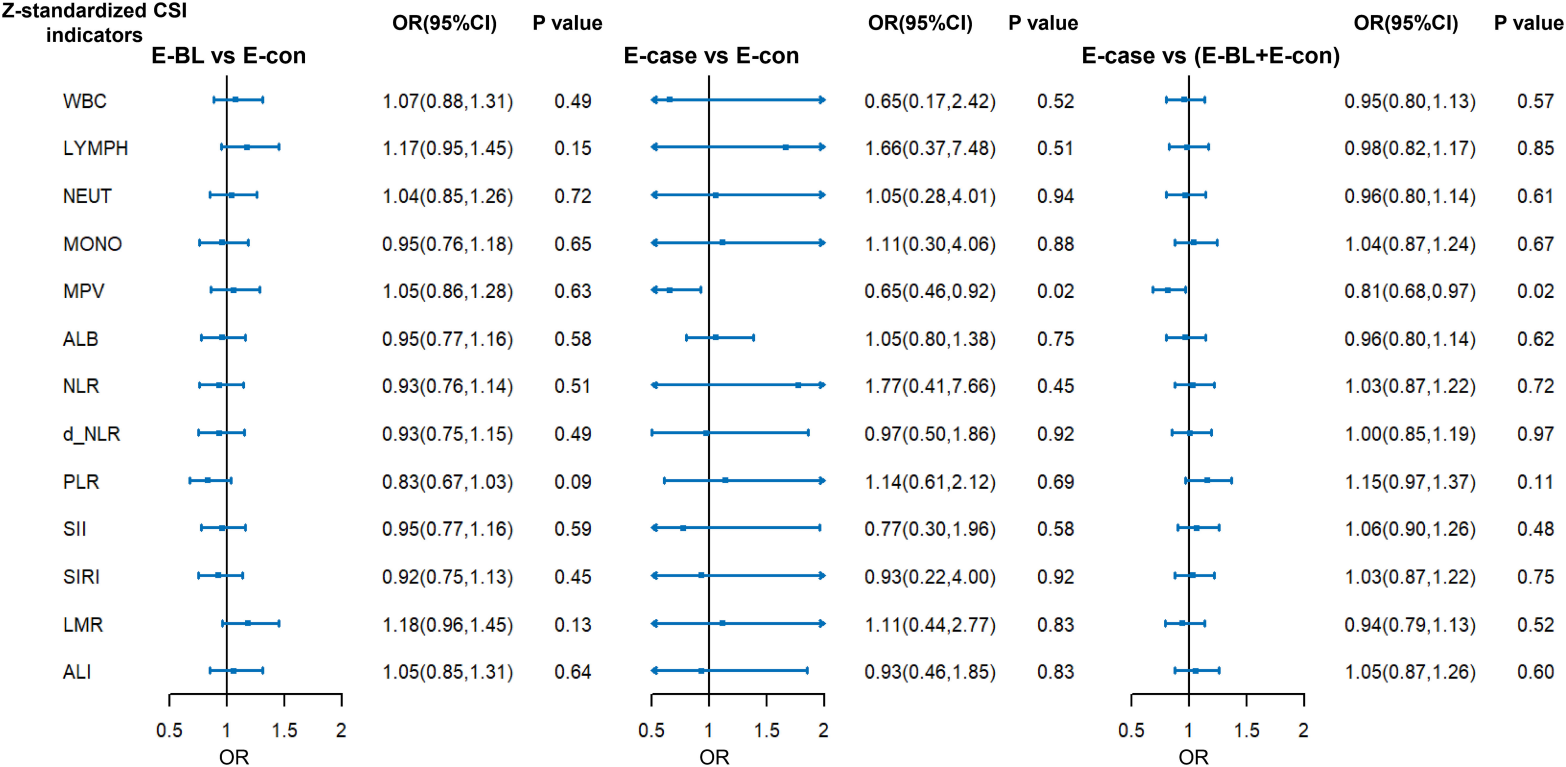
The forest plots of the associations of CSI indicators and esophageal lesions in the case-control studies of esophageal lesions.

Conditional logistic regression models were constructed to explore the association between CSI indicators and esophageal precancerous lesion among participants. When compared to E-con group, none of 13 CSI indicators was associated with esophageal benign lesion (E-BL group) with non-significant ORs (**Figure 2**, **Supplementary Table 1**), while, only the lower levels of Z-standardized MPV was associated with higher risk of esophageal precancerous lesion/cancer (E-case group) with an OR (95%CI) of 0.65 (0.46, 0.92) (*P* = 0.015) (**Figure 2**, **Supplementary Table 2**), after adjusting for age, sex, alcohol drinking and smoking status, education, marriage, occupation, BMI, family wealth score, physical activity, and family history of cancer. When combined the E-case and E-BL groups and compared with E-con group, none of the ORs for 13 CSI indicators were statistically significant (**Supplementary Table 3**).

When compared E-case to E-BL groups, the OR (95%CI) was 0.82 (0.67, 1.00) (*P* = 0.053), and 1.28 (1.04,1.58) (*P* = 0.020) for MPV and PLR in fully adjusted unconditional logistic regression models (**Supplementary Table 4**). Considering the severity of the lesions, we reclassified E-BL and E-con groups as a whole control group. The OR of MPV remained statistically significant of 0.81(0.68, 0.97) (*P* = 0.022) for precancerous lesion/cancer risk in the fully adjusted unconditional logistic regression model (**Figure 2**, **Supplementary Table 5**).

### 3.3 The association between CSI and gastric lesions

The levels of 13 CSI indicators in the different stages of gastric lesions groups are summarized in **Supplementary Table 6**. None of the 13 CSI indicators exhibited statistically significant differences among the G-con, AG, IM and G-case groups. When compared in pairs, only WBC and MONO were statistically higher in the G-case group than AG group.

**Figure 3:**
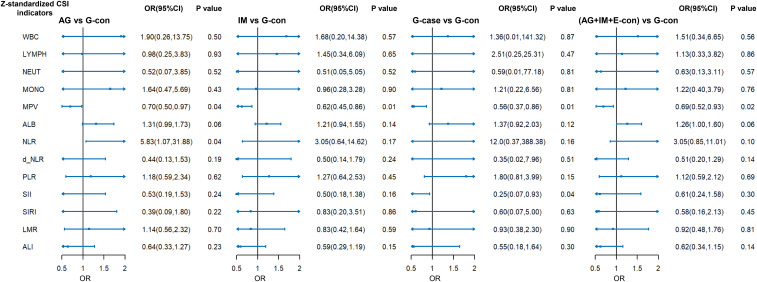
The forest plots of the associations of CSI indicators and gastric lesions in case-control studies of gastric lesions.

In conditional logistic regression analysis of case-control study, we set G-con as control group and AG, IM or G-case as case group, respectively. The ORs (95%CI) of MPV were 0.70 (0.50,0.97) (*P* = 0.039), 0.62 (0.45,0.86) (*P* = 0.005), or 0.56 (0.37,0.86) (*P* = 0.008) in fully adjusted models (**Figure 3**; **Supplementary Tables 7**-**9**). According to the Correa’s cascade pathway of progression to GC, we combined AG, IM and G-case into a gastric lesion group as case group, and the corresponding OR (95%CI) of MPV was 0.69 (0.52,0.93) (*P* = 0.015) in model 3 with G-con as control group (**Figure 3**, **Supplementary Table 10**).

To further explore the changing trend of MPV from AG, IM to G-case group, case-control studies were conducted in pairs, and the results of conditional logistic regression models are shown in **Supplementary Tables 11**-**13**. None of ORs of CSI indicators were significant in fully adjusted models. In addition, considering the nature of lesions, we compared G-case group to all other lesion groups (G-con, AG and IM), the ORs of 13 CSI indicators were still non-significant in fully-adjusted regression models (**Supplementary Table 14**).

## 4 Discussion

The persistent inflammatory microenvironment could accelerate tumor progression (22). Thus, we summarized 13 currently reported CSI indicators and explored their potential roles in multi-stage lesions of the esophagus and stomach. In the case-control study of esophageal lesions, we observed a slight decrease in MPV level from normal mucosa or benign lesion to precancerous lesion/cancer, and MPV was inversely associated with the risk of esophageal precancerous lesion/cancer. In gastric lesions, MPV was also inversely associated with the risk of AG and more severe lesions in Correa’s cascade pathway of GC, indicating MPV decrease appears early in the cascade, but did not further decrease when the lesion severity develops.

Platelets are anucleate cells released into the bloodstream following the cytoplasmic cleavage of megakaryocytes. Their volumes are heterogeneous, and MPV has been used as a precise assessment of their dimensions, typically ranging from 7.5 to 12.0 fl. Generally, larger platelet means higher platelet activity, and large platelets should account for 0.2-5.0% of the total population (23). The feedback system in human body maintains the balance of platelet count and MPV in healthy individuals. However, during ongoing inflammation, this balance is disrupted and increased large platelets are released in the stimulation of intracellular synthesis of procoagulant and proinflammatory factors, degranulation of granules, and initiation of the platelet pool in the spleen. They firstly aggregate at the site of injury, activating fibrosis and inflammatory processes (24). Furthermore, MPV is accessible in routine hematological analyzer, thus its clinical significance in the progression of various pathological conditions has received extensive attention, such as cardiovascular diseases, respiratory diseases, diabetes mellitus, and so on (23).

Recently, a systematic review investigated the potential role of MPV in 12 types of cancer, and recommended it as a promising tool for disease diagnosis and optimization of therapeutic strategies (25). In ESCC, low MPV has been observed in cancer patients and is associated with advanced tumor length and cancer-specific survival (26, 27). Conversely, higher MPV has also been reported in ESCC (28, 29). However, most of these studies were based on clinically diagnosed ESCC, which may lead to a reversal causality. Therefore, we conducted the case-control study among population diagnosed after sample collection and involved those with benign lesions. We supported the negative association of MPV with ESCC, based on its negative correlation with the risk of esophageal benign lesions and precancerous lesion/cancer. From esophagitis to precancerous lesion, inflammatory response is persistent from which could activate the coagulation system, causing a large number of platelets to aggregate at the site of injury. Meanwhile, pro-inflammatory factors inhibit the proliferation and maturation of megakaryocytes in the bone marrow, impede their cytoplasmic extension and the formation of platelet precursors, resulting in smaller platelets being released into the peripheral blood. These lead to the decrease of MPV in patients with inflammation, especially acute inflammation (30). As the inflammatory response intensifies, larger platelet, which are more metabolically and enzymatically active than the smaller, continuously generate in the altered megakaryocyte-platelet haemostatic axis, which explained the high levels in clinically diagnosed ESCC patients (28, 29). We hypothesize that MPV exhibits a biphasic pattern: a gradual decline during tumor initiation, followed by a subsequent increase upon malignant transformation. However, this trend warrants further validation through prospective studies.

Correa’s cascade is a classic pathological process in the development of GC, in which active chronic inflammation plays a crucial functional role (31). Previous studies have reported a close association between various CSI indicators and diagnosed GC (8–11, 32, 33). Differently, our current study included subjects with multi-stage lesions within Correa’s cascade of GC, revealing that only MPV was negatively associated with the risk of AG and more serious lesions, even precancerous lesion/cancer, when compared to SG/normal control groups. A previous case-control study also reported lower MPV levels in GC and IM than healthy controls (34). However, it contrasts with the results of other retrospective case-control studies, in which MPV levels in GC patients were higher than or similar with those in healthy individuals (32, 33, 35). Combing all these, we hypothesize that MPV may play a more important role in the initial stages of gastric mucosal inflammation rather than in the later stages of disease progression. In future studies, we need to conduct prospective follow-up to verify this hypothesis, and more specific inflammatory markers should be detected to identify the key indicators of inflammation in the development of GC.

The primary strength of our current study was the complete description of CSI levels across multi-stage pathological lesions in the development of ESCC and GC, derived from a natural population-based screening program, in which all information and samples were collected before the diagnosis of lesions. Our study design focuses on the benign lesion and precancerous stages in esophagus and stomach, and avoided the reverse causality bias in diagnosed cancer patients. However, there are some limitations that need to be addressed. First, our case-control study design necessitates further prospective cohort validation to draw robust conclusions about the association of CSI with the development of EC or GC. Second, our study was conducted in a high-incidence area of China, which limits the generalizability of the results to other populations in China or other countries. Third, various factors may affect CSI levels, including aging, lifestyle, and metabolic disorders. The risk of residual confounding is inevitable, although a series of factors were adjusted in our analysis. Fourth, *H. pylori* infection is a significant risk factor for gastric lesions [36], but it was not discussed currently, which should be included in further studies. Last, we summarized all currently reported easy-to-detect CSI markers in our analysis, but more indicators, such as interleukin, tumor necrosis factor, C-reactive protein, and others [37, 38], should also be measured to provide a more comprehensive description on the role of inflammation in the development of ESCC and GC.

In summary, our current study found that a low MPV was associated with an increased risk of precancerous lesion/cancer in the esophagus. Differently, decreased MPV was associated with higher risk of early-stage gastric lesions, including AG and more severe lesions, in Correa’s cascade pathway of GC. Further in-depth exploration in larger prospective studies based on more specific indicators of inflammation is necessary.

## Data Availability

The raw data supporting the conclusions of this article will be made available by the authors, without undue reservation upon reasonable request.
