# Supplementary material for: Levels of chronic systemic inflammation markers in patients with multi-stages of esophageal and gastric lesions
Source: Front Oncol. 2026 Jul 8;16:1751801. doi: 10.3389/fonc.2026.1751801 (PMC13389850; doi:10.3389/fonc.2026.1751801)
Supplement: Supplementary file 1 [file Table1.docx]

Supplementary Material

**Supplemental Table 1.** Unconditional logistic regression analysis of the association between CSI and esophageal benign lesion compared with normal control group

| **CSI indicators** | **Model 1** | | **Model 2** | | **Model 3** | |
| --- | --- | --- | --- | --- | --- | --- |
|  | **OR (95%CI)** | ***P*** | **OR (95%CI)** | ***P*** | **OR (95%CI)** | ***P*** |
| WBC | 1.08(0.90,1.31) | 0.410 | 1.08(0.89,1.31) | 0.420 | 1.07(0.88,1.31) | 0.489 |
| LYMPH | 1.19(0.98,1.45) | 0.086 | 1.18(0.96,1.44) | 0.110 | 1.17(0.95,1.45) | 0.151 |
| NEUT | 1.05(0.87,1.27) | 0.595 | 1.05(0.86,1.27) | 0.639 | 1.04(0.85,1.26) | 0.722 |
| MONO | 0.91(0.74,1.11) | 0.345 | 0.95(0.77,1.17) | 0.634 | 0.95(0.76,1.18) | 0.645 |
| MPV | 1.01(0.84,1.22) | 0.914 | 1.04(0.86,1.25) | 0.723 | 1.05(0.86,1.28) | 0.631 |
| ALB | 0.96(0.79,1.16) | 0.647 | 0.94(0.77,1.14) | 0.517 | 0.95(0.77,1.16) | 0.580 |
| NLR | 0.92(0.76,1.12) | 0.422 | 0.93(0.76,1.13) | 0.450 | 0.93(0.76,1.14) | 0.510 |
| d_NLR | 0.92(0.75,1.13) | 0.426 | 0.92(0.75,1.12) | 0.402 | 0.93(0.75,1.15) | 0.490 |
| PLR | 0.84(0.69,1.03) | 0.090 | 0.83(0.68,1.02) | 0.078 | 0.83(0.67,1.03) | 0.090 |
| SII | 0.95(0.78,1.16) | 0.620 | 0.94(0.77,1.15) | 0.563 | 0.95(0.77,1.16) | 0.589 |
| SIRI | 0.89(0.74,1.09) | 0.254 | 0.92(0.75,1.12) | 0.398 | 0.92(0.75,1.13) | 0.448 |
| LMR | 1.23(1.01,1.49) | 0.037 | 1.18(0.97,1.44) | 0.105 | 1.18(0.96,1.45) | 0.128 |
| ALI | 1.09(0.90,1.33) | 0.374 | 1.07(0.88,1.31) | 0.481 | 1.05(0.85,1.31) | 0.641 |

Note: All CSI indicators are Z-standardized before analysis.

The outcome was E-BL (N=229) group compared with normal control group (N=202).

Model 1 was unadjusted. Model 2 was adjusted for age and sex. Model 3 was adjusted for age and sex, drinking and smoking status, education, marriage, occupation, BMI, family wealth score, physical activity, and family history of cancer.

**Supplemental Table 2.** Conditional logistic regression analysis of the association between CSI and esophageal precancerous lesion/cancer compared with normal control group

| **CSI indicators** | **Model 1** | | **Model 2** | | **Model 3** | |
| --- | --- | --- | --- | --- | --- | --- |
|  | **OR (95%CI)** | ***P*** | **OR (95%CI)** | ***P*** | **OR (95%CI)** | ***P*** |
| WBC | 0.61(0.18,2.06) | 0.422 | 0.63(0.18,2.17) | 0.465 | 0.65(0.17,2.42) | 0.516 |
| LYMPH | 2.02(0.55,7.37) | 0.287 | 1.97(0.54,7.20) | 0.307 | 1.66(0.37,7.48) | 0.512 |
| NEUT | 1.05(0.31,3.50) | 0.940 | 1.03(0.31,3.47) | 0.956 | 1.05(0.28,4.01) | 0.942 |
| MONO | 0.80(0.26,2.50) | 0.700 | 0.82(0.26,2.57) | 0.730 | 1.11(0.30,4.06) | 0.877 |
| MPV | 0.72(0.53,0.99) | 0.041 | 0.71(0.52,0.97) | 0.034 | 0.65(0.46,0.92) | 0.015 |
| ALB | 0.93(0.73,1.19) | 0.585 | 0.96(0.75,1.22) | 0.720 | 1.05(0.80,1.38) | 0.749 |
| NLR | 1.19(0.33,4.29) | 0.787 | 1.40(0.38,5.12) | 0.610 | 1.77(0.41,7.66) | 0.445 |
| d_NLR | 0.98(0.54,1.79) | 0.954 | 0.96(0.53,1.77) | 0.905 | 0.97(0.50,1.86) | 0.921 |
| PLR | 1.04(0.58,1.85) | 0.897 | 1.00(0.56,1.77) | 0.990 | 1.14(0.61,2.12) | 0.691 |
| SII | 0.96(0.41,2.21) | 0.916 | 0.89(0.39,2.07) | 0.795 | 0.77(0.30,1.96) | 0.580 |
| SIRI | 1.25(0.36,4.39) | 0.726 | 1.13(0.32,3.99) | 0.847 | 0.93(0.22,4.00) | 0.920 |
| LMR | 0.86(0.38,1.93) | 0.712 | 0.82(0.36,1.85) | 0.626 | 1.11(0.44,2.77) | 0.832 |
| ALI | 0.87(0.53,1.42) | 0.569 | 0.88(0.54,1.43) | 0.595 | 0.93(0.46,1.85) | 0.825 |

Note: All CSI indicators are Z-standardized before analysis.

The outcome was E-case (N=202) group compared with normal group (N=202) by matched age and sex in 1:1 ratio.

Model 1 was unadjusted. Model 2 was adjusted for age and sex. Model 3 was adjusted for age and sex, drinking and smoking status, education, marriage, occupation, BMI, family wealth score, physical activity, and family history of cancer.

**Supplemental Table 3.** Unconditional logistic regression analysis of the association between CSI and esophageal benign lesion/precancerous lesion/cancer compared with normal control group

| **CSI indicators** | **Model 1** | | **Model 2** | | **Model 3** | |
| --- | --- | --- | --- | --- | --- | --- |
|  | **OR (95%CI)** | ***P*** | **OR (95%CI)** | ***P*** | **OR (95%CI)** | ***P*** |
| WBC | 1.03(0.87,1.22) | 0.702 | 1.04(0.88,1.23) | 0.668 | 1.04(0.87,1.24) | 0.699 |
| LYMPH | 1.11(0.93,1.32) | 0.239 | 1.11(0.93,1.31) | 0.250 | 1.11(0.93,1.32) | 0.270 |
| NEUT | 1.02(0.86,1.20) | 0.833 | 1.02(0.86,1.21) | 0.834 | 1.01 (0.85,1.21) | 0.873 |
| MONO | 0.97(0.82,1.14) | 0.669 | 0.99(0.83,1.18) | 0.899 | 0.99(0.83,1.18) | 0.886 |
| MPV | 0.92(0.78,1.09) | 0.344 | 0.93(0.79,1.10) | 0.405 | 0.94(0.79,1.11) | 0.463 |
| ALB | 0.93(0.79,1.10) | 0.385 | 0.92(0.78,1.09) | 0.340 | 0.94(0.79,1.12) | 0.483 |
| NLR | 0.97(0.82,1.14) | 0.687 | 0.97(0.82,1.14) | 0.702 | 0.97(0.82,1.15) | 0.723 |
| d_NLR | 0.95(0.81,1.13) | 0.572 | 0.95(0.80,1.13) | 0.552 | 0.95(0.80,1.13) | 0.533 |
| PLR | 0.96(0.81,1.13) | 0.589 | 0.95(0.80,1.12) | 0.523 | 0.94(0.80,1.12) | 0.507 |
| SII | 1.00(0.85,1.18) | 0.990 | 1.00(0.84,1.18) | 0.951 | 0.99(0.83,1.18) | 0.919 |
| SIRI | 0.95(0.80,1.12) | 0.521 | 0.96(0.81,1.14) | 0.651 | 0.96(0.81,1.14) | 0.671 |
| LMR | 1.11(0.94,1.32) | 0.213 | 1.09(0.91,1.30) | 0.335 | 1.10(0.91,1.31) | 0.329 |
| ALI | 1.05(0.89,1.24) | 0.584 | 1.04(0.88,1.23) | 0.660 | 1.05(0.87,1.26) | 0.611 |

Note: All CSI indicators are Z-standardized before analysis.

The outcome was E-case/benign (N=431) group compared with normal control group (N=202).

Model 1 was unadjusted. Model 2 was adjusted for age and sex. Model 3 was adjusted for age and sex, drinking and smoking status, education, marriage, occupation, BMI, family wealth score, physical activity, and family history of cancer.

**Supplemental Table 4.** Unconditional logistic regression analysis of the association between CSI and esophageal precancerous lesion/cancer compared with benign lesion group

| **CSI indicators** | **Model 1** | | **Model 2** | | **Model 3** | |
| --- | --- | --- | --- | --- | --- | --- |
|  | **OR (95%CI)** | ***P*** | **OR (95%CI)** | ***P*** | **OR (95%CI)** | ***P*** |
| WBC | 0.90(0.74,1.09) | 0.291 | 0.89(0.73,1.08) | 0.226 | 0.91(0.74,1.11) | 0.345 |
| LYMPH | 0.88(0.73,1.06) | 0.187 | 0.88(0.73,1.06) | 0.169 | 0.93(0.76,1.13) | 0.445 |
| NEUT | 0.93(0.77,1.13) | 0.460 | 0.93(0.77,1.13) | 0.448 | 0.93(0.76,1.13) | 0.465 |
| MONO | 1.11(0.92,1.34) | 0.279 | 1.06(0.87,1.29) | 0.582 | 1.05(0.86,1.28) | 0.658 |
| MPV | 0.82(0.68,1.00) | 0.044 | 0.81(0.67,0.99) | 0.036 | 0.82(0.67,1.00) | 0.053 |
| ALB | 0.94(0.78,1.13) | 0.505 | 0.95(0.78,1.15) | 0.585 | 0.98(0.80,1.19) | 0.819 |
| NLR | 1.089(0.903,1.314) | 0.372 | 1.09(0.91,1.32) | 0.358 | 1.05(0.87,1.28) | 0.602 |
| d_NLR | 1.050(0.877,1.257) | 0.593 | 1.06(0.88,1.27) | 0.526 | 1.03(0.86,1.25) | 0.737 |
| PLR | 1.28(1.05,1.55) | 0.016 | 1.33(1.08,1.63) | 0.007 | 1.28(1.04,1.58) | 0.020 |
| SII | 1.10(0.91,1.32) | 0.318 | 1.12(0.93,1.35) | 0.233 | 1.09(0.90,1.32) | 0.375 |
| SIRI | 1.12(0.93,1.36) | 0.242 | 1.09(0.90,1.32) | 0.385 | 1.05(0.86,1.28) | 0.625 |
| LMR | 0.81(0.67,0.99) | 0.035 | 0.84(0.69,1.03) | 0.094 | 0.89(0.73,1.10) | 0.289 |
| ALI | 0.92(0.76,1.12) | 0.412 | 0.93(0.77,1.13) | 0.489 | 1.05(0.85,1.30) | 0.667 |

Note: All CSI indicators are Z-standardized before analysis.

The outcome was E-case (N=202) group compared with E-BL group (N=229).

Model 1 was unadjusted. Model 2 was adjusted for age and sex. Model 3 was adjusted for age and sex, drinking and smoking status, education, marriage, occupation, BMI, family wealth score, physical activity, and family history of cancer.

**Supplemental Table 5.** Unconditional logistic regression analysis of the association between CSI and esophageal precancerous lesion/cancer compared with benign/normal control group

| **CSI indicators** | **Model 1** | | **Model 2** | | **Model 3** | |
| --- | --- | --- | --- | --- | --- | --- |
|  | **OR (95%CI)** | ***P*** | **OR (95%CI)** | ***P*** | **OR (95%CI)** | ***P*** |
| WBC | 0.94(0.79,1.11) | 0.456 | 0.93(0.79,1.11) | 0.425 | 0.95(0.80,1.13) | 0.565 |
| LYMPH | 0.95(0.80,1.12) | 0.520 | 0.95(0.80,1.12) | 0.536 | 0.98(0.82,1.17) | 0.854 |
| NEUT | 0.95(0.81,1.13) | 0.579 | 0.95(0.80,1.13) | 0.575 | 0.96(0.80,1.14) | 0.613 |
| MONO | 1.07(0.90,1.26) | 0.443 | 1.04(0.88,1.24) | 0.624 | 1.04(0.87,1.24) | 0.674 |
| MPV | 0.83(0.70,0.98) | 0.027 | 0.82(0.69,0.97) | 0.020 | 0.81(0.68,0.97) | 0.022 |
| ALB | 0.92(0.78,1.09) | 0.319 | 0.92(0.78,1.09) | 0.352 | 0.96(0.80,1.14) | 0.615 |
| NLR | 1.05(0.89,1.24) | 0.540 | 1.05(0.89,1.24) | 0.553 | 1.03(0.87,1.22) | 0.722 |
| d_NLR | 1.02(0.86,1.21) | 0.814 | 1.02(0.86,1.21) | 0.791 | 1.00(0.85,1.19) | 0.968 |
| PLR | 1.17(0.99,1.38) | 0.060 | 1.18(1.00,1.40) | 0.048 | 1.15(0.97,1.37) | 0.106 |
| SII | 1.08(0.92,1.27) | 0.360 | 1.09(0.92,1.28) | 0.322 | 1.06(0.90,1.26) | 0.476 |
| SIRI | 1.06(0.90,1.25) | 0.462 | 1.05(0.89,1.24) | 0.581 | 1.03(0.87,1.22) | 0.750 |
| LMR | 0.89(0.75,1.06) | 0.190 | 0.91(0.76,1.09) | 0.296 | 0.94(0.79,1.13) | 0.519 |
| ALI | 0.96(0.81,1.14) | 0.645 | 0.97(0.82,1.15) | 0.722 | 1.05(0.87,1.26) | 0.603 |

Note: All CSI indicators are Z-standardized before analysis.

The outcome was E-case (N=202) group compared with benign/normal control group (E-BL+E-con, N=431).

Model 1 was unadjusted. Model 2 was adjusted for age and sex. Model 3 was adjusted for age and sex, drinking and smoking status, education, marriage, occupation, BMI, family wealth score, physical activity, and family history of cancer.

**Supplement Table 6.** The chronic systemic inflammation levels of different stages of gastric lesions

| **CSI Indicators** | **Overall**  **(N=624)** | **G-con**  **(N=156)** | **AG**  **(N=156)** | **IM**  **(N=156)** | **G-case**  **(N=156)** | ***P* value** | **P value of subgroup analysis** | | | | | |
| --- | --- | --- | --- | --- | --- | --- | --- | --- | --- | --- | --- | --- |
|  |  |  |  |  |  |  | **AG *vs* G-con** | **IM *vs* G-con** | **G-case *vs* G-con** | **IM *vs* AG** | **G-case *vs* AG** | **G-case *vs* IM** |
| WBC, 10^9^/L | 6.14(5.24,7.13) | 6.14(5.20,6.95) | 5.98(5.20,6.95) | 6.20(5.21,7.24) | 6.34(5.36,7.36) | 0.133 | 0.272 | 0.504 | 0.251 | 0.659 | 0.028 | 0.076 |
| LYMPH, 10^9^/L | 2.06(1.72,2.48) | 2.06(1.71,2.45) | 2.01(1.71,2.41) | 2.08(1.72,2.51) | 2.08(1.75,2.58) | 0.482 | 0.409 | 0.609 | 0.520 | 0.787 | 0.146 | 0.242 |
| NEUT, 10^9^/L | 3.45(2.79,4.22) | 3.32(2.78,4.25) | 3.42(2.76,4.05) | 3.49(2.78,4.31) | 3.57(2.90,4.30) | 0.425 | 0.384 | 0.516 | 0.552 | 0.868 | 0.144 | 0.171 |
| MONO, 10^9^/L | 0.37(0.30,0.47) | 0.38(0.30,0.47) | 0.35(0.29,0.44) | 0.38(0.31,0.47) | 0.38(0.32,0.49) | 0.215 | 0.266 | 0.709 | 0.347 | 0.141 | 0.041 | 0.562 |
| MPV, fL | 10.50(9.65,11.20) | 10.50(9.55,11.20) | 10.40(9.70,11.30) | 10.60(9.90,11.30) | 10.50(9.50,11.10) | 0.465 | 0.364 | 0.175 | 0.160 | 0.656 | 0.617 | 0.871 |
| ALB, g/L | 47.70(45.55,49.50) | 47.75(45.45,49.55) | 47.60(45.85,49.40) | 47.60(45.55,49.55) | 47.80(45.45,49.45) | 0.865 | 0.382 | 0.653 | 0.717 | 0.733 | 0.640 | 0.847 |
| NLR | 1.69(1.32,2.10) | 1.69(1.32,2.08) | 1.71(1.25,2.15) | 1.65(1.32,2.11) | 1.69(1.35,2.04) | 0.988 | 0.929 | 0.865 | 0.713 | 0.917 | 0.816 | 0.880 |
| d_NLR | 1.32(1.05,1.61) | 1.33(1.07,1.63) | 1.34(1.00,1.62) | 1.30(1.04,1.63) | 1.32(1.08,1.58) | 0.967 | 0.697 | 0.892 | 0.683 | 0.863 | 0.833 | 0.724 |
| PLR | 113.00(88.67,140.58) | 118.28(90.35,140.81) | 114.64(89.61,141.75) | 107.45(87.34,141.33) | 109.73(88.18,138.98) | 0.780 | 0.707 | 0.460 | 0.875 | 0.770 | 0.540 | 0.317 |
| LMR | 5.54(4.40,6.81) | 5.51(4.22,6.41) | 5.59(4.55,6.89) | 5.56(4.48,7.02) | 5.48(4.38,6.79) | 0.554 | 0.787 | 0.322 | 0.562 | 0.176 | 0.390 | 0.646 |
| SII | 385.32(283.50,532.90) | 379.21(284.37,513.69) | 391.75(277.74,535.47) | 380.57(280.02,558.21) | 386.97(298.39,516.34) | 0.971 | 0.890 | 0.949 | 0.751 | 0.833 | 0.628 | 0.786 |
| SIRI | 0.63(0.45,0.87) | 0.64(0.44,0.87) | 0.60(0.44,0.84) | 0.63(0.45,0.90) | 0.64(0.48,0.87) | 0.622 | 0.575 | 0.679 | 0.492 | 0.349 | 0.203 | 0.788 |
| ALI | 691.64(525.31,885.35) | 685.86(524.34,857.00) | 696.26(541.43,902.57) | 691.64(531.32,896.81) | 688.95(515.87,871.73) | 0.865 | 0.688 | 0.846 | 0.697 | 0.546 | 0.399 | 0.837 |

**Supplemental Table 7.** Conditional logistic regression analysis of the association between CSI and gastric AG compared with SG/normal control group

| **CSI indicators** | **Model 1** | | **Model 2** | | **Model 3** | |
| --- | --- | --- | --- | --- | --- | --- |
|  | **OR (95%CI)** | ***P*** | **OR (95%CI)** | ***P*** | **OR (95%CI)** | ***P*** |
| WBC | 1.31(0.03,62.64) | 0.892 | 0.97(0.02,44.14) | 0.986 | 1.90(0.26,13.75) | 0.499 |
| LYMPH | 0.76(0.16,3.65) | 0.727 | 0.84(0.18,4.00) | 0.828 | 0.98(0.25,3.83) | 0.926 |
| NEUT | 1.59(0.03,85.32) | 0.819 | 2.10(0.04,107.71) | 0.713 | 0.52(0.07,3.85) | 0.524 |
| MONO | 1.60(0.45,5.76) | 0.470 | 1.70(0.47,6.13) | 0.418 | 1.64(0.47,5.69) | 0.430 |
| MPV | 0.75(0.56,1.01) | 0.054 | 0.75(0.56,1.00) | 0.053 | 0.70(0.50,0.97) | 0.039 |
| ALB | 1.11(0.87,1.41) | 0.391 | 1.13(0.88,1.43) | 0.339 | 1.31(0.99,1.73) | 0.060 |
| NLR | 6.71(0.43,103.99) | 0.173 | 7.93(0.50,124.91) | 0.141 | 5.83(1.07,31.88) | 0.040 |
| d_NLR | 0.31(0.02,4.08) | 0.374 | 0.27(0.02,3.54) | 0.316 | 0.44(0.13,1.53) | 0.186 |
| PLR | 1.31(0.68,2.52) | 0.418 | 1.33(0.69,2.56) | 0.399 | 1.18(0.59,2.34) | 0.621 |
| SII | 0.49(0.18,1.33) | 0.162 | 0.48(0.18,1.31) | 0.152 | 0.53(0.19,1.53) | 0.243 |
| SIRI | 0.27(0.05,1.41) | 0.122 | 0.26(0.05,1.36) | 0.110 | 0.39(0.09,1.80) | 0.218 |
| LMR | 0.93(0.48,1.82) | 0.837 | 0.95(0.49,1.85) | 0.881 | 1.14(0.56,2.32) | 0.702 |
| ALI | 1.08(0.66,1.75) | 0.762 | 1.07(0.66,1.73) | 0.792 | 0.64(0.33,1.27) | 0.229 |

Note: All CSI indicators are Z-standardized before analysis.

The outcome was gastric AG group (N=156) compared with matched SG/normal control group (N=156).

Model 1 was unadjusted. Model 2 was adjusted for age and sex. Model 3 was adjusted for age and sex, drinking and smoking status, education, marriage, occupation, BMI, family wealth score, physical activity, family history of cancer, and *H. pylori* infection status.

**Supplemental Table 8.** Conditional logistic regression analysis of the association between CSI and gastric IM compared with SG/normal control group

| **CSI indicators** | **Model 1** | | **Model 2** | | **Model 3** | |
| --- | --- | --- | --- | --- | --- | --- |
|  | **OR (95%CI)** | ***P*** | **OR (95%CI)** | ***P*** | **OR (95%CI)** | ***P*** |
| WBC | 1.35(0.05,38.54) | 0.861 | 1.35(0.05,38.54) | 0.861 | 1.68(0.20,14.38) | 0.570 |
| LYMPH | 0.93(0.21,4.01) | 0.917 | 0.93(0.21,4.01) | 0.917 | 1.45(0.34,6.09) | 0.648 |
| NEUT | 1.30(0.04,45.73) | 0.886 | 1.30(0.04,45.73) | 0.886 | 0.51(0.05,5.05) | 0.524 |
| MONO | 1.13(0.35,3.71) | 0.839 | 1.13(0.35,3.71) | 0.839 | 0.96(0.28,3.28) | 0.902 |
| MPV | 0.69(0.51,0.93) | 0.014 | 0.69(0.51,0.93) | 0.014 | 0.62(0.45,0.86) | 0.005 |
| ALB | 1.14(0.91,1.43) | 0.252 | 1.14(0.91,1.43) | 0.252 | 1.21(0.94,1.55) | 0.140 |
| NLR | 2.97(0.33,26.58) | 0.330 | 2.97(0.33,26.58) | 0.330 | 3.05(0.64,14.62) | 0.166 |
| d_NLR | 0.45(0.05,3.84) | 0.462 | 0.45(0.05,3.84) | 0.462 | 0.50(0.14,1.79) | 0.241 |
| PLR | 1.37(0.70,2.66) | 0.356 | 1.37(0.70,2.66) | 0.356 | 1.27(0.64,2.53) | 0.449 |
| SII | 0.46(0.17,1.22) | 0.120 | 0.46(0.17,1.22) | 0.120 | 0.50(0.18,1.38) | 0.163 |
| SIRI | 0.60(0.150,2.37) | 0.462 | 0.60(0.15,2.37) | 0.462 | 0.83(0.20,3.51) | 0.856 |
| LMR | 0.83(0.43,1.59) | 0.567 | 0.83(0.43,1.59) | 0.567 | 0.83(0.42,1.64) | 0.591 |
| ALI | 0.94(0.58,1.53) | 0.809 | 0.94(0.58,1.53) | 0.809 | 0.59(0.29,1.19) | 0.150 |

Note: All CSI indicators are Z-standardized before analysis.

The outcome was gastric IM group (N=156) compared with matched SG/normal control group (N=156).

Model 1 was unadjusted. Model 2 was adjusted for age and sex. Model 3 was adjusted for age and sex, drinking and smoking status, education, marriage, occupation, BMI, family wealth score, physical activity, family history of cancer, and *H. pylori* infection status.

**Supplemental Table 9.** Conditional logistic regression analysis of the association between CSI and gastric precancerous lesion/cancer compared with SG/normal control group

| **CSI indicators** | **Model 1** | | **Model 2** | | **Model 3** | |
| --- | --- | --- | --- | --- | --- | --- |
|  | **OR (95%CI)** | ***P*** | **OR (95%CI)** | ***P*** | **OR (95%CI)** | ***P*** |
| WBC | 0.82(0.02,37.84) | 0.917 | 0.82(0.02,37.84) | 0.917 | 1.36(0.01,141.32) | 0.869 |
| LYMPH | 1.52(0.26,9.06) | 0.645 | 1.52(0.26,9.06) | 0.645 | 2.51(0.25,25.31) | 0.468 |
| NEUT | 2.14(0.04,122.22) | 0.712 | 2.14(0.04,122.22) | 0.712 | 0.59(0.01,77.18) | 0.814 |
| MONO | 1.27(0.29,5.55) | 0.747 | 1.27(0.29,5.55) | 0.747 | 1.21(0.22,6.56) | 0.809 |
| MPV | 0.66(0.46,0.94) | 0.021 | 0.66(0.46,0.94) | 0.021 | 0.56(0.37,0.86) | 0.008 |
| ALB | 1.14(0.84,1.55) | 0.403 | 1.14(0.84,1.55) | 0.403 | 1.37(0.92,2.03) | 0.118 |
| NLR | 9.50(0.44,206.63) | 0.152 | 9.50(0.44,206.63) | 0.152 | 12.0(0.37,388.38) | 0.157 |
| d_NLR | 0.23(0.01,3.67) | 0.295 | 0.23(0.01,3.67) | 0.295 | 0.35(0.02,7.96) | 0.508 |
| PLR | 1.58(0.76,3.28) | 0.217 | 1.58(0.76,3.28) | 0.217 | 1.80(0.81,3.99) | 0.147 |
| SII | 0.33(0.10,1.06) | 0.062 | 0.33(0.10,1.06) | 0.062 | 0.25(0.07,0.93) | 0.038 |
| SIRI | 0.44(0.07,2.74) | 0.376 | 0.44(0.07,2.74) | 0.376 | 0.60(0.07,5.00) | 0.625 |
| LMR | 0.84(0.38,1.87) | 0.665 | 0.84(0.38,1.87) | 0.665 | 0.93(0.38,2.30) | 0.898 |
| ALI | 0.89(0.47,1.69) | 0.711 | 0.89(0.47,1.69) | 0.711 | 0.55(0.18,1.64) | 0.297 |

Note: All CSI indicators are Z-standardized before analysis.

The outcome was G-case (N=156) group compared with matched SG/normal control group (N=156).

Model 1 was unadjusted. Model 2 was adjusted for age and sex. Model 3 was adjusted for age and sex, drinking and smoking status, education, marriage, occupation, BMI, family wealth score, physical activity, family history of cancer, and *H. pylori* infection status.

**Supplemental Table 10.** Conditional logistic regression analysis of the association between CSI and gastric lesion compared with SG/normal control group

| **CSI indicators** | **Model 1** | | **Model 2** | | **Model 3** | |
| --- | --- | --- | --- | --- | --- | --- |
|  | **OR (95%CI)** | ***P*** | **OR (95%CI)** | ***P*** | **OR (95%CI)** | ***P*** |
| WBC | 1.53(0.09,27.13) | 0.771 | 1.32(0.04,39.33) | 0.872 | 1.51(0.34,6.65) | 0.564 |
| LYMPH | 0.77(0.21,2.81) | 0.687 | 0.81(0.20,3.30) | 0.764 | 1.13(0.33,3.82) | 0.861 |
| NEUT | 1.22(0.06,24.49) | 0.899 | 1.39(0.04,48.19) | 0.855 | 0.63(0.13,3.11) | 0.568 |
| MONO | 1.21(0.40,3.67) | 0.743 | 1.24(0.39,3.93) | 0.710 | 1.22(0.40,3.79) | 0.756 |
| MPV | 0.73(0.56,0.96) | 0.023 | 0.73(0.56,0.96) | 0.022 | 0.69(0.52,0.93) | 0.015 |
| ALB | 1.14(0.92,1.41) | 0.242 | 1.15(0.92,1.43) | 0.221 | 1.26(1.00,1.60) | 0.055 |
| NLR | 3.15(0.48,20.67) | 0.233 | 3.39(0.39,29.34) | 0.267 | 3.05(0.85,11.01) | 0.096 |
| d_NLR | 0.46(0.08,2.72) | 0.391 | 0.43(0.05,3.54) | 0.432 | 0.51(0.20,1.29) | 0.142 |
| PLR | 1.27(0.68,2.38) | 0.460 | 1.28(0.68,2.40) | 0.449 | 1.12(0.59,2.12) | 0.694 |
| SII | 0.52(0.21,1.32) | 0.171 | 0.52(0.21,1.31) | 0.166 | 0.61(0.24,1.58) | 0.302 |
| SIRI | 0.46(0.13,1.69) | 0.245 | 0.45(0.12,1.75) | 0.250 | 0.58(0.16,2.13) | 0.447 |
| LMR | 0.84(0.45,1.55) | 0.568 | 0.84(0.45,1.57) | 0.592 | 0.92(0.48,1.76) | 0.808 |
| ALI | 1.02(0.65,1.61) | 0.919 | 1.02(0.65,1.60) | 0.931 | 0.62(0.34,1.15) | 0.140 |

Note: All CSI indicators are Z-standardized before analysis.

The outcome was gastric lesions group (N=468, including G-case, AG, and IM group) compared with SC/normal group (N=156).

Model 1 was unadjusted. Model 2 was adjusted for age and sex. Model 3 was adjusted for age and sex, drinking and smoking status, education, marriage, occupation, BMI, family wealth score, physical activity, family history of cancer, and *H. pylori* infection status.

**Supplemental Table 11.** Conditional logistic regression analysis of the association between CSI and gastric IM compared with AG group

| **CSI indicators** | **Model 1** | | **Model 2** | | **Model 3** | |
| --- | --- | --- | --- | --- | --- | --- |
|  | **OR (95 %CI)** | ***P*** | **OR (95 %CI)** | ***P*** | **OR (95 %CI)** | ***P*** |
| WBC | 1.24(0.45,3.41) | 0.683 | 1.32(0.47,3.71) | 0.597 | 1.57(0.49,5.08) | 0.447 |
| LYMPH | 1.17(0.37,3.67) | 0.794 | 1.13(0.36,3.58) | 0.834 | 0.87(0.22,3.42) | 0.829 |
| NEUT | 0.74(0.23,2.41) | 0.622 | 0.72(0.22,2.36) | 0.582 | 0.72(0.17,3.07) | 0.664 |
| MONO | 0.77(0.21,2.77) | 0.683 | 0.75(0.21,2.73) | 0.659 | 0.70(0.16,3.00) | 0.646 |
| MPV | 0.88(0.67,1.16) | 0.380 | 0.89(0.68,1.17) | 0.411 | 0.88(0.65,1.19) | 0.404 |
| ALB | 0.97(0.77,1.23) | 0.826 | 0.95(0.75,1.21) | 0.681 | 0.95(0.73,1.25) | 0.718 |
| NLR | 0.75(0.23,2.43) | 0.629 | 0.76(0.23,2.49) | 0.654 | 0.97(0.26,3.62) | 0.969 |
| d_NLR | 0.83(0.47,1.46) | 0.510 | 0.79(0.44,1.43) | 0.440 | 0.72(0.37,1.41) | 0.353 |
| PLR | 1.19(0.61,2.33) | 0.604 | 1.18(0.60,2.31) | 0.631 | 1.28(0.61,2.66) | 0.516 |
| SII | 0.66(0.22,1.92) | 0.440 | 0.67(0.23,1.95) | 0.458 | 0.54(0.16,1.79) | 0.313 |
| SIRI | 2.68(0.57,12.68) | 0.213 | 2.66(0.56,12.64) | 0.218 | 2.87(0.46,17.97) | 0.270 |
| LMR | 1.00(0.46,2.15) | 0.991 | 0.99(0.46,2.14) | 0.974 | 0.98(0.43,2.24) | 0.966 |
| ALI | 0.75(0.45,1.25) | 0.271 | 0.76(0.46,1.27) | 0.289 | 1.09(0.50,2.38) | 0.835 |

Note: All CSI indicators are Z-standardized before analysis.

The outcome was gastric IM group (N=156) compared with matched AG group (N=156).

Model 1 was unadjusted. Model 2 was adjusted for age and sex. Model 3 was adjusted for age and sex, drinking and smoking status, education, marriage, occupation, BMI, family wealth score, physical activity, family history of cancer, and *H. pylori* infection status.

**Supplemental Table 12.** Conditional logistic regression analysis of the association between CSI and gastric precancerous lesion/cancer compared with AG group

| CSI indicators | Model 1 | | Model 2 | | Model 3 | |
| --- | --- | --- | --- | --- | --- | --- |
|  | OR (95%CI) | *P* | OR (95%CI) | *P* | OR (95%CI) | *P* |
| WBC | 2.06(0.54,7.87) | 0.291 | 2.32(0.58,9.22) | 0.233 | 3.33(0.59,18.64) | 0.173 |
| LYMPH | 0.94(0.24,3.69) | 0.933 | 0.89(0.22,3.49) | 0.862 | 0.81(0.13,4.99) | 0.827 |
| NEUT | 0.65(0.16,2.58) | 0.541 | 0.62(0.15,2.49) | 0.497 | 0.32(0.05,2.15) | 0.242 |
| MONO | 0.92(0.22,3.90) | 0.906 | 0.89(0.21,3.81) | 0.875 | 0.88(0.14,5.62) | 0.890 |
| MPV | 0.89(0.64,1.24) | 0.480 | 0.89(0.64,1.23) | 0.473 | 0.86(0.58,1.27) | 0.435 |
| ALB | 0.94(0.71,1.25) | 0.671 | 0.91(0.68,1.21) | 0.507 | 0.96(0.68,1.37) | 0.821 |
| NLR | 1.12(0.24,5.26) | 0.885 | 1.21(0.25,5.74) | 0.813 | 2.00(0.30,13.29) | 0.472 |
| d_NLR | 0.63(030,1.34) | 0.233 | 0.60(0.28,1.29) | 0.189 | 0.42(0.16,1.15) | 0.091 |
| PLR | 1.31(0.62,2.78) | 0.485 | 1.28(0.60,2.72) | 0.526 | 1.30(0.52,3.22) | 0.581 |
| SII | 0.60(0.17,2.06) | 0.414 | 0.60(0.17,2.07) | 0.418 | 0.5(0.12,2.44) | 0.437 |
| SIRI | 2.11(0.33,13.46) | 0.430 | 2.01(0.314,12.93) | 0.460 | 2.29(0.22,23.81) | 0.488 |
| LMR | 1.08(0.45,2.60) | 0.864 | 1.06(0.44,2.56) | 0.893 | 1.12(0.393.27) | 0.831 |
| ALI | 0.81(0.42,1.57) | 0.534 | 0.84(0.43,1.62) | 0.598 | 0.78(0.29,2.15) | 0.635 |

Note: All CSI indicators are Z-standardized before analysis.

The outcome was G-case (N=156) group compared with matched AG group (N=156).

Model 1 was unadjusted. Model 2 was adjusted for age and sex. Model 3 was adjusted for age and sex, drinking and smoking status, education, marriage, occupation, BMI, family wealth score, physical activity, family history of cancer, and *H. pylori* infection status.

**Supplemental Table 13.** Conditional logistic regression analysis of the association between CSI and gastric precancerous lesion/cancer compared with IM group

| **CSI indicators** | **Model 1** | | **Model 2** | | **Model 3** | |
| --- | --- | --- | --- | --- | --- | --- |
|  | **OR (95%CI)** | ***P*** | **OR (95%CI)** | ***P*** | **OR (95%CI)** | ***P*** |
| WBC | 0.57(0.02,17.14) | 0.746 | 0.57(0.02,17.14) | 0.746 | 0.50(0.01,17.99) | 0.824 |
| LYMPH | 1.42(0.29,6.83) | 0.665 | 1.42(0.29,6.83) | 0.665 | 1.62(0.27,9.61) | 0.593 |
| NEUT | 3.45(0.07,181.73) | 0.540 | 3.45(0.07,181.73) | 0.540 | 3.66(0.05,259.06) | 0.673 |
| MONO | 1.10(0.27,4.48) | 0.895 | 1.10(0.27,4.48) | 0.895 | 1.09(0.23,5.27) | 0.931 |
| MPV | 1.00(0.72,1.39) | 0.997 | 1.00(0.72,1.39) | 0.997 | 1.07(0.75,1.53) | 0.739 |
| ALB | 1.02(0.79,1.33) | 0.868 | 1.02(0.79,1.33) | 0.868 | 0.99(0.74,1.34) | 0.954 |
| NLR | 3.53(0.26,48.43) | 0.345 | 3.53(0.26,48.43) | 0.345 | 4.22(0.25,70.09) | 0.381 |
| d_NLR | 0.34(0.02,5.39) | 0.440 | 0.34(0.02,5.39) | 0.440 | 0.25(0.01,4.75) | 0.392 |
| PLR | 1.53(0.71,3.27) | 0.276 | 1.53(0.71,3.27) | 0.276 | 1.44(0.66,3.17) | 0.316 |
| SII | 0.52(0.16,1.69) | 0.278 | 0.52(0.16,1.69) | 0.278 | 0.59(0.17,2.02) | 0.381 |
| SIRI | 0.68(0.12,3.73) | 0.655 | 0.68(0.12,3.73) | 0.655 | 0.66(0.09,4.80) | 0.837 |
| LMR | 1.06(0.45,2.47) | 0.896 | 1.06(0.45,2.47) | 0.896 | 1.05(0.43,2.59) | 0.986 |
| ALI | 1.02(0.55,1.91) | 0.945 | 1.02(0.55,1.91) | 0.945 | 0.86(0.34,2.17) | 0.670 |

Note: All CSI indicators are Z-standardized before analysis.

The outcome was G-case (N=156) group compared with matched IM group (N=156).

Model 1 was unadjusted. Model 2 was adjusted for age and sex. Model 3 was adjusted for age and sex, drinking and smoking status, education, marriage, occupation, BMI, family wealth score, physical activity, family history of cancer, and *H. pylori* infection status.

**Supplemental Table 14.** Conditional logistic regression analysis of the association between CSI and gastric precancerous lesion/cancer compared with other lesion and SG/normal control group

| **CSI indicators** | **Model 1** | | **Model 2** | | **Model 3** | |
| --- | --- | --- | --- | --- | --- | --- |
|  | **OR (95 %CI)** | ***P*** | **OR (95%CI)** | ***P*** | **OR (95%CI)** | ***P*** |
| WBC | 2.04(0.83,5.04) | 0.121 | 2.08(0.849,5.15) | 0.114 | 2.21(0.80,6.07) | 0.118 |
| LYMPH | 1.03(0.38,2.79) | 0.961 | 1.01(0.37,2.76) | 0.978 | 1.13(0.36,3.55) | 0.857 |
| NEUT | 0.75(0.27,2.09) | 0.584 | 0.75(0.27,2.09) | 0.583 | 0.60(0.18,1.93) | 0.375 |
| MONO | 0.87(0.30,2.49) | 0.788 | 0.86(0.30,2.47) | 0.773 | 0.83(0.27,2.59) | 0.734 |
| MPV | 0.85(0.65,1.11) | 0.225 | 0.85(0.65,1.11) | 0.229 | 0.86(0.65,1.12) | 0.274 |
| ALB | 1.01(0.81,1.25) | 0.935 | 1.00(0.81,1.25) | 0.976 | 1.04(0.82,1.32) | 0.759 |
| NLR | 1.82(0.62,5.35) | 0.276 | 1.81(0.62,5.32) | 0.282 | 2.04(0.66,6.29) | 0.209 |
| d_NLR | 0.61(0.36,1.04) | 0.068 | 0.61(0.36,1.03) | 0.065 | 0.56(0.31,1.02) | 0.052 |
| PLR | 1.43(0.82,2.51) | 0.207 | 1.42(0.81,2.49) | 0.216 | 1.45(0.80,2.62) | 0.212 |
| SII | 0.50(0.20,1.20) | 0.120 | 0.50(0.21,1.21) | 0.125 | 0.50(0.20,1.28) | 0.145 |
| SIRI | 1.06(0.32,3.50) | 0.919 | 1.06(0.32,3.50) | 0.924 | 1.14(0.31,4.20) | 0.824 |
| LMR | 0.92(0.47,1.78) | 0.799 | 0.91(0.47,1.77) | 0.784 | 0.96(0.48,1.92) | 0.921 |
| ALI | 0.87(0.53,1.41) | 0.558 | 0.87(0.54,1.41) | 0.573 | 0.73(0.37,1.44) | 0.376 |

Note: All CSI indicators are Z-standardized before analysis.

The outcome was G-case (N=156) group compared with matched other lesion group (N=468), including IM (N=156), AG (N=156), and SG/normal (N=156).

Model 1 was unadjusted. Model 2 was adjusted for age and sex. Model 3 was adjusted for age and sex, drinking and smoking status, education, marriage, occupation, BMI, family wealth score, physical activity, family history of cancer, and *H. pylori* infection status.
